# The 4MOTHERS trial of the impact of a mobile money-based intervention on maternal and neonatal health outcomes in Madagascar: study protocol of a cluster-randomized hybrid effectiveness-implementation trial

**DOI:** 10.1186/s13063-021-05694-8

**Published:** 2021-10-21

**Authors:** Etienne Lacroze, Till Bärnighausen, Jan Walter De Neve, Sebastian Vollmer, Rolland Marie Ratsimbazafy, Peter Martin Ferdinand Emmrich, Nadine Muller, Elsa Rajemison, Zavaniarivo Rampanjato, Diana Ratsiambakaina, Samuel Knauss, Julius Valentin Emmrich

**Affiliations:** 1 Heidelberg Institute of Global Health, Medical Faculty and University Hospital, University of Heidelberg, Heidelberg, Germany; 2 Global Digital Health Lab, Charité - Universitätsmedizin Berlin, Berlin, Germany; 3 Department of Global Health and Population, Harvard T.H. Chan School of Public Health, Boston, Massachusetts USA; 4 Africa Health Research Institute (AHRI), Mtubatuba, KwaZulu-Natal South Africa; 5 Department of Economics and Centre for Modern Indian Studies, University of Göttingen, Göttingen, Germany; 6 National Institute of Public and Community Health, Antananarivo, Madagascar; 7 John Innes Centre, Norwich, UK; 8 Department of Infectious Diseases and Respiratory Medicine, Charité - Universitätsmedizin Berlin, Berlin, Germany; 9 Ministry of Public Health of the Republic of Madagascar, Antananarivo, Madagascar; 10 Charité Global Health and Department of Experimental Neurology and Center for Stroke Research, Charité - Universitätsmedizin Berlin, Charitéplatz 1, 10117 Berlin, Germany; 11 Berlin Institute of Health, Berlin, Germany

**Keywords:** Maternal, Out-of-pocket payments, Randomized trial, Digital health, Mobile payment, Sub-Saharan Africa, Universal health coverage

## Abstract

**Background:**

Mobile money—a service enabling users to receive, store, and send electronic money using mobile phones—has been widely adopted across low- and middle-income economies to pay for a variety of services, including healthcare. However, evidence on its effects on healthcare access and health outcomes are scarce and the possible implications of using mobile money for financing and payment of maternal healthcare services—which generally require large one-time out-of-pocket payments—have not yet been systematically assessed in low-resource settings. The aim of this study is to determine the impact on health outcomes, cost-effectiveness, feasibility, acceptability, and usefulness of mobile phone-based savings and payment service, the Mobile Maternal Health Wallet (MMHW), for skilled healthcare during pregnancy and delivery among women in Madagascar.

**Methods:**

This is a hybrid effectiveness-implementation type-1 trial, determining the effectiveness of the intervention while evaluating the context of its implementation in Madagascar’s Analamanga region, containing the capital, Antananarivo. Using a stratified cluster randomized design, 61 public-sector primary-care health facilities were randomized within 6 strata to either receive the intervention or not (29 intervention vs. 32 control facilities). The strata were defined by a health facility’s antenatal care visit volume and its capacity to offer facility-based deliveries. The registered pre-specified primary outcomes are (i) delivery at a health facility, (ii) antenatal care visits, and (iii) total healthcare expenditure during pregnancy, delivery, and neonatal period. The registered pre-specified secondary outcomes include additional health outcomes, economic outcomes, and measurements of user experience and satisfaction. Our estimated enrolment number is 4600 women, who completed their pregnancy between July 1, 2020, and December 31, 2021. A series of nested mixed-methods studies will elucidate client and provider perceptions on feasibility, acceptability, and usefulness of the intervention to inform future implementation efforts.

**Discussion:**

A cluster-randomized, hybrid effectiveness-implementation design allows for a robust approach to determine whether the MMHW is a feasible and beneficial intervention in a resource-restricted public healthcare environment. We expect the results of our study to guide future initiatives and health policy decisions related to maternal and neonatal health and universal healthcare coverage through technology in Madagascar and other countries in sub-Saharan Africa.

**Trial registration:**

This trial was registered on March 12, 2021: Deutsches Register Klinischer Studien (German Clinical Trials Register), identifier: DRKS00014928. For World Health Organization Trial Registration Data Set see Additional file 1.

**Supplementary Information:**

The online version contains supplementary material available at 10.1186/s13063-021-05694-8.

## Background

Despite the widespread introduction of user-fee exemption policies to improve access to skilled care during pregnancy and childbirth, out-of-pocket (OOP) payments remain the predominant mode for households in sub-Saharan Africa (SSA) to cater for the costs of healthcare [1]. However, direct costs, including consultations, diagnostic procedures, medicines, and surgery, and indirect costs, such as transportation to a health facility or time off work and associated loss of income, often exceed the available assets and savings of a low-income household [2–6]. Assets such as agricultural land or livestock are usually difficult to convert into cash immediately. In addition, many poor households have no assured regular income or collateral to secure a loan, thus preventing insurance companies and financial institutions from providing their services to these potentially high-risk customers [7]. Therefore, every year 1.5% of the population in SSA are pushed into extreme poverty due to catastrophic health expenditure [8, 9]. To prevent medical impoverishment, households depend on remittances from families and friends, turn to borrowing from formal and informal financial institutions and cut back on non-healthcare expenses such as education and food [1, 2]. Besides, household savings can help to protect from the financial burden of unexpected health expenditure [1, 5]. However, poor households often face the need for unexpected and irregular expenses, which may undercut long-term saving goals [10]. In consequence, expectant mothers from poor households in SSA often do not seek skilled care during pregnancy and childbirth to avoid the risk of being driven deeper into poverty [11]. This is further compounded by the widespread disruption of maternal healthcare services due to the COVID-19 pandemic [12].

Within less than a generation, mobile communication has become ubiquitous. During the last 10 years, mobile phone ownership more than doubled in low- and middle-income countries (LMICs) and quadrupled in SSA [13]. Today, almost 80% of worldwide mobile phone subscriptions come from LMICs with more than 75 subscriptions per 100 people in SSA, whereas, at least until 2025, SSA is expected to achieve the strongest growth of mobile phone penetration among world regions [13, 14]. Alongside this mobile revolution followed payment services, also known as mobile money (MM). MM enables users to receive, save, and send electronic money on a digital platform run by a mobile operator, acting as an alternative to cash. MM can also be used to pay for utility bills or goods and services or to receive bulk payments such as salaries. Using low tech such as unstructured supplementary service data (USSD), MM services can be designed to not require a data connection or smartphone capabilities, thus making them suitable for virtually any mobile handset. Cash can be deposited at agents or retail stores and converted into MM and vice versa [15]. Since 2011, MM increased the overall access to financial services in SSA from 23% in 2011 to 43% in 2017 illustrating the financial inclusion of households with low transaction volumes or limited geographical access which are otherwise underserved by the formal banking system [16]. By late 2019, MM services were available in 95 countries with almost half of all MM users being registered in SSA, accounting for around two thirds of the global MM transaction volume [17]. MM frequently allows users to also enroll in additional financial services, including savings and credit schemes or insurances offered by mobile operators or third-party providers. In addition, MM allows users to perform financial transactions with minimal physical contact, which may be beneficial to curb the transmission of infectious diseases.

Owing to its potential to provide users with rapid access to cash and remittances, electronic savings accounts, and insurance schemes, MM is increasingly being used in the health sector in SSA [17]. MM users have an overall lower risk of catastrophic health expenditure during emergency care and are less likely to reduce non-medical expenses for education or food than non-users [18–22]. Services employing MM in the context of public and private healthcare systems have been launched in several countries in the region, including for (micro)insurance schemes [23–26], loans [27], electronic saving platforms [28, 29], and conditional cash transfers [30]. These services have had mixed results. While the Kenyan National Health Insurance Fund has thrived since the introduction of MM-payable premiums [25], a number of smaller services have been taken off the market due to low demand or active-user conversion rate [24, 28, 31]. Among the multitude of medical conditions, maternal healthcare seems particularly well suited for a savings scheme. Specifically, expenses for maternal healthcare are largely predictable both in their timing and amount. However, the potential benefits and implications of using MM and related services for financing and payment of maternal medical expenses have not yet been systematically assessed.

### Madagascar

In Madagascar, an island nation with a population of 25 million and one of the least developed countries worldwide, financial obstacles are a major cause of limited access to skilled maternal healthcare [32, 33]. Only around half of the pregnant women in Madagascar complete 4 antenatal care (ANC) visits as recommended by the World Health Organization (WHO) and 54% of deliveries take place without qualified personnel [32, 34]. The maternal mortality rate in 2017 was 335 per 100,000 live births and was estimated to be up to 3 times higher in the poorest districts of the country [35, 36]. OOP payments represent 24.7% of total healthcare expenditure and despite national efforts to implement universal health coverage (UHC) using mobile technology, there are currently no MM-based dedicated services for pregnancy-related savings, health insurance, or direct cash transfers available in Madagascar. The risk for impoverishing health expenses during pregnancy is high [37–39]. However, within the last decade, mobile phone subscription rates have increased from less than 3 subscriptions per 100 people in 2005 to 40 per 100 in 2018 [13]. The footsteps of this mobile revolution have followed MM services. As a result, MM accounts have overtaken the number of formal bank accounts in Madagascar in 2015 [16]. In preparation for this trial, we conducted a human-centered, mixed-methods design study in Antananarivo, the capital of the island, to determine the structural, contextual, and experiential characteristics of a mobile phone-based savings and payment service for skilled healthcare during pregnancy and delivery. We demonstrated a high degree of perceived usefulness of MM-based savings and payments for maternal healthcare among key stakeholders and women, in particular among those from low-income households [40, 41].

### Conceptual framework and study objectives

We hypothesize that the implementation of a MM-based payment and savings service for maternal healthcare, the so-called Mobile Maternal Health Wallet (MMHW), will improve access to skilled care during pregnancy and childbirth by reducing financial obstacles. To test this hypothesis, we designed a hybrid effectiveness-implementation type-1 trial, called the Madagascar Mobile MOney for maTernal HEalthcare-Related Spending (4MOTHERS) trial, for evaluation of the MMHW in public-sector health facilities in the Analamanga region of Madagascar. Our trial complements the randomized implementation of the MMHW intervention by a non-governmental organization. The reason for facility randomization was to ensure procedural equality as the implementer’s funding constraints limited the number of facilities allocated to the intervention. The 4MOTHERS trial will adopt a multidisciplinary, cluster-randomized approach complemented by a process oriented mixed-methods evaluation to assess the impact of the intervention on (i) maternal and neonatal health outcomes, (ii) financial outcomes, and (iii) its feasibility, acceptability, and usefulness in the context of its implementation. More generally, the trial will contribute to our understanding of the health system impact of a digital tool for financial inclusion and access to essential healthcare services. In doing so, the trial will add to the scarce evidence on the usefulness of digital tools to achieve UHC. The evidence from this trial is important because it can inform governments in SSA on how to improve access to essential healthcare services, and maternal healthcare in particular, in public-sector healthcare systems.

### Primary and secondary outcomes

Our primary outcomes are (i) woman delivering at a health facility (facility-based delivery), (ii) ANC visits at a health facility per woman (ANC visits), and (iii) total health expenditure during pregnancy, delivery, and neonatal period (total healthcare expenditure).

Our secondary outcomes are as follows:

(i) Maternal and newborn health outcomes include the following:

- Pregnancy or childbirth-related diagnoses and complications
- Postpartum depression
- Maternal and neonatal mortality

(ii) Economic outcomes include the following:

- Remittances received from relatives and friends
- Relative household health expenditure
- Implementer costs and public sector costs of the intervention
- Cost-effectiveness of the intervention for ANC and facility-based delivery
- Participants’ financial distress due to maternal and neonatal healthcare costs

(iii) Measurements of participants’ and healthcare providers’ experience, satisfaction, and behavior include the following:

- Time to seek medical attention
- MMHW usage
- User and health system satisfaction

A complete list of registered and unregistered outcomes for this trial is shown in Table 1.

**Table 1:** Primary and secondary outcomes of the 4MOTHERS trial

Outcomes	Definition	Variable/unit	Data source
**Primary outcomes**			
Facility-based delivery^a^	Woman delivering at a health facility	Binary variable	Quantitative household survey
ANC visits^a^	ANC visits at a health facility per woman	Count variable	Quantitative household survey
Total healthcare expenditure^a^	Total health expenditure during pregnancy, delivery and neonatal period per woman	Continuous variable	Quantitative household survey, mmhw dashboard
**Secondary outcomes**			
ANC diagnoses^a^	Diagnoses detected during ANC per woman	Count variable	Quantitative household survey
Complications^a^	Pregnancy or childbirth-related complications	Count variable	Quantitative household survey
Postpartum depression^a^	Women interviewed after delivery fulfilling the screening criteria for depression	Edinburgh self-reported Postnatal Depression Scale	Quantitative household survey
Maternal mortality^a^	Maternal deaths	Binary variable	Quantitative household survey, health facility records
Newborn mortality^a^	Newborn deaths	Binary variable	Quantitative household survey, health facility records
Elective C-section	Women reporting a referral for an elective C-section	Binary variable	Quantitative household survey
Emergency C-section	Emergency C-section referrals	Binary variable	Quantitative household survey
Prenatal ultrasound examination	Woman receiving a prenatal ultrasound examination	Binary variable	Quantitative household survey
ANC drugs received	Woman receiving iron and folic acid supplements during ANC	Binary variable	Quantitative household survey
ANC drugs distributed	Iron and folic acid supplements distributed	Continuous variable	Health facility records
Third parties’ financial contributions^a^	Funds received from relatives and friends for maternal and neonatal healthcare	Continuous variable	Quantitative household survey, MMHW dashboard
Health savings	Total savings for maternal and neonatal healthcare per woman	Continuous variable	Quantitative household survey
Relative healthcare expenditure^a^	Ratio of total healthcare expenditure of household income	Ratio	Quantitative household survey
Direct costs of the intervention	Amount spent by the implementer for the development and implementation of the MMHW	Continuous variable	Implementer records
Implementer’s healthcare contributions	Healthcare expenditure contributed by implementer per woman using conditional cash transfers or electronic vouchers	Continuous variable	MMHW dashboard
Public sector costs^a^	Healthcare expenditure per woman during pregnancy, delivery and neonatal period	Continuous variable	Health facility records
Cost per additional facility-based delivery^a^	Cost-effectiveness ratio: effect on the first primary outcome divided by effect on the third primary outcome	Ratio	Quantitative household survey, MMHW dashboard
Cost per additional ANC visit^a^	Cost-effectiveness ratio: effect on the second primary outcome divided by effect on the third primary outcome	Ratio	Quantitative household survey, MMHW dashboard
Financial distress^a^	Woman reporting at least one sign of financial distress	Binary variable	Quantitative household survey
Time to seek medical attention^a^	Time from first symptoms until professional medical care was accessed	Continuous time variable	Quantitative household survey
MMHW usage^a^	Woman who used the MMHW to pay for ANC or delivery-related expenses either in part or entirely	Binary variable	Quantitative household survey
Patient satisfaction^a^	Patient satisfaction with health facility and MMHW	Likert scale, qualitative interviews	Quantitative and qualitative household survey
Health system satisfaction^a^	Satisfaction with the health system	Likert scale, qualitative interviews	Quantitative and qualitative household survey
Life satisfaction	Satisfaction with general life situation	Likert scale, qualitative interviews	Quantitative and qualitative household survey
Time of sign-up	Time of sign-up to MMHW in relation to delivery date	Continuous time variable	Quantitative household survey, MMHW dashboard

^a^Pre-registered outcomes at DRKS (Deutsches Register Klinischer Studien, German Clinical Trials Register, identifier: DRKS00014928); *MMHW* maternal mobile health wallet, *ANC* antenatal care, *C-section* caesarean section

## Methods

### Study setting

We will conduct this study in the health districts Antananarivo North (Avaradrano), Centre (Renivohitra), and South (Atsimondrana) within the region of Analamanga, Madagascar. The districts are urban, peri-urban, and rural and include Antananarivo, the capital of the country. The study region has 2.2 million inhabitants, 31% of whom live under the national poverty line (535,603 Malagasy Ariary per capita income; 248.42 USD, 2011 PPP), considerably less than the Malagasy average (71%) [32, 42]; 77.4% of the Malagasy population live below the international poverty line of 1.9$ per day (2011 PPP) [43]. The lingua franca is Malagasy. In the study region Analamanga, 65% of pregnant women complete at least 4 ANC visits, 68% deliver at a public or private health facility while 74% have qualified assistance during birth [34]. Public healthcare in these districts is provided by 65 public-sector health facilities, comprising 61 primary-care health facilities (“Centre de santé de base”) and four public reference hospitals for maternal healthcare. Primary-care health facility staff includes at least one doctor and two midwives or nurses. Each primary-care health facility has approximately 15–30 affiliated community health workers (CHWs) who advise pregnant women to seek ANC at the primary-care health facility [44]. In addition, CHWs provide information on general health aspects, possible complications, and nutrition during pregnancy. Each of the primary-care health facilities in the study region performed between 40 and 5437 prenatal examinations and 0 to 1150 deliveries in 2017. If complications occur or more specific treatment is required, primary-care health facilities can refer patients, at the patient’s expense, to a reference hospital. ANC, vaginal and C-section deliveries as well as accommodation are free of charge for patients at primary-care health facilities and reference hospitals. However, any medication, lab tests or materials required for delivery or surgery must be purchased in cash by patients from the pharmacy, which is affiliated with a health facility. A vaginal delivery without complications costs on average about 12 USD and a C-section costs around 128 USD in total, representing 3% and 32% of the average annual salary in the region, respectively [45–47].

### Study design

We will conduct this trial comparing the intervention package to the usual standard of care in 61 public-sector primary-care health facilities—randomized by 6 strata—in Madagascar’s Analamanga region. We selected a hybrid effectiveness-implementation type-1 design, because it blends design components of a cluster-randomized impact evaluation and mixed-methods evaluation to inform future implementation efforts [48]. This trial complements an intervention which was co-created with the Malagasy Ministry of Health and implemented by a non-governmental organization. The motivation for facility randomization was the need to ration the intervention to selected facilities due to the implementer’s funding constraints. Randomization allowed for the unbiased allocation of facilities to the intervention or control group free from political or logistical reasons while ensuring procedural equality [49]. After intervention implementation, it became clear that a scientific evaluation would be feasible allowing for strong causal inference of the effect of the intervention. Thus, trial registration occurred after randomization and intervention implementation on March 12, 2021, but before the initiation of outcome assessment. This manuscript complies with the SPIRIT checklist (see Additional file 2) [50].

### Eligibility criteria

#### Clusters

**Fig. 1:**
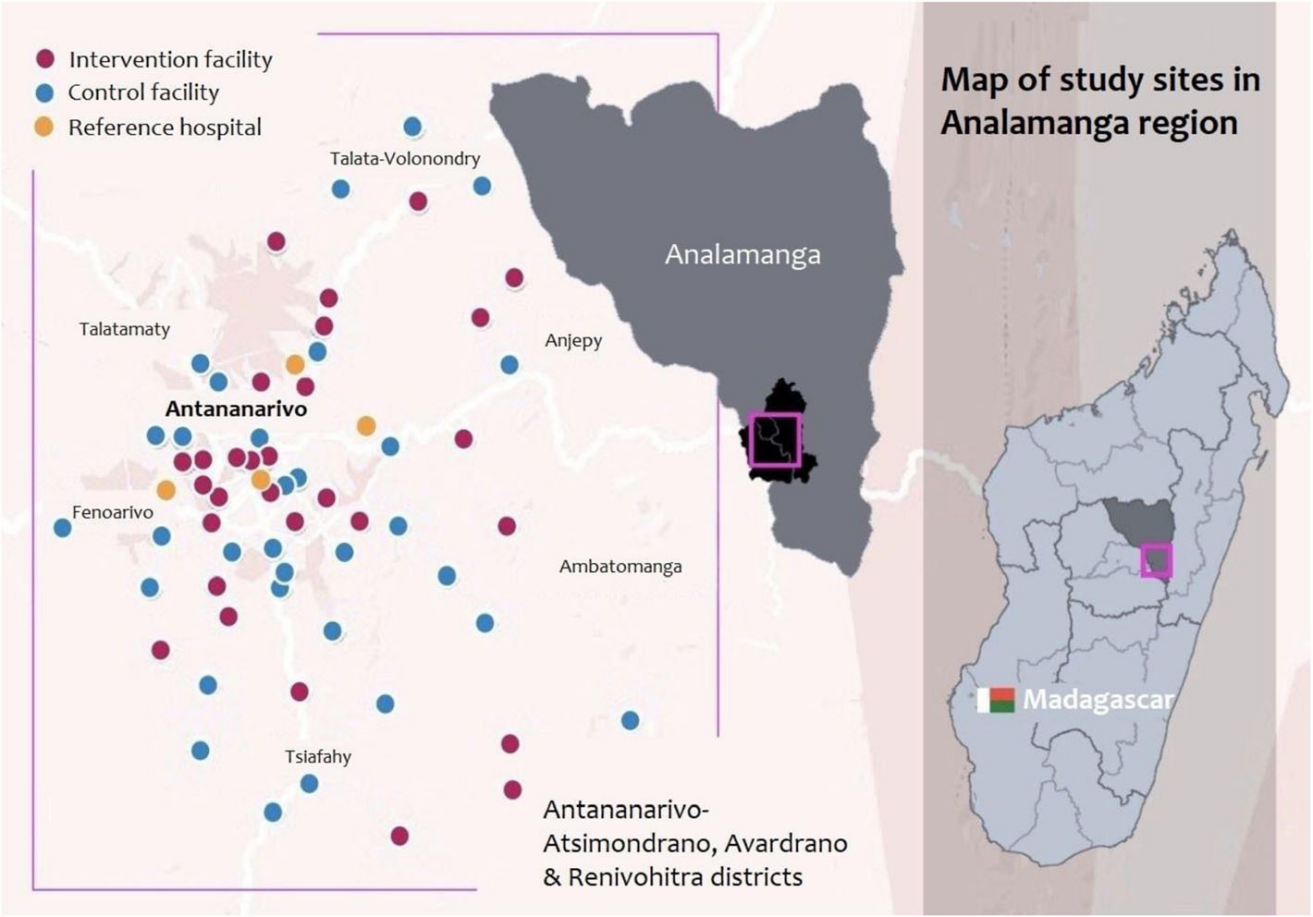
Public-sector primary-care health facilities and reference hospitals in Antananarivo Atsimondrana (South), Renivohitra (Centre), and Avaradrano (North) health districts in Analamanaga region, Madagascar

All public-sector primary-care health facilities that perform ANC in the study region were eligible for randomization (Fig. 1).

#### Inclusion criteria

All young mothers who lived in a randomly selected census enumeration area within the catchment area of a primary-care health facility participating in this study and who completed their pregnancy between July 1, 2020, and December 31, 2021, will be asked for their verbal consent to participate in the study. Participants must be able and willing to give verbal consent for trial participation; they must be at least 18 years old to participate. Providers will be eligible to be included in the study if they are working within a facility where the study is taking place and are at least 18 years old.

#### Exclusion criteria

Individuals considered unable to provide and participate in informed consent including those with uncontrolled psychiatric disorders or severe neurological impairment resulting in an inability to participate in the informed consent process.

### Procedures

#### Intervention package

The intervention package examined in this study consists of three elements: (i) the MMHW for restricted health savings during pregnancy, (ii) conditional cash transfers and electronic vouchers for maternal and neonatal healthcare services and emergency evacuation, and (iii) quality of care assessments and trainings for facility-based health workers. The intervention package is implemented by a non-governmental organization in partnership with the Malagasy Ministry of Health’s UHC program. Sequential implementation of the intervention package commenced in January 2019 and all randomized facilities received the intervention at least since May 2020.

#### Mobile Maternal Health Wallet

The core system behind the MMHW is a software that allows users to pay for maternal healthcare services using MM at participating health facilities in return for treatment-related data being provided by the health facility to the MMHW service provider. On the user’s side, the MMHW is an unstructured supplementary service data (USSD) menu, which is accessible through the Global System for Mobile communication networks (GSM) of the two major Malagasy mobile phone operators by dialing a 3-digit number followed by the hash sign. Thus, using the MMHW does not require an active internet connection or smartphone capabilities. Users can save to the MMHW using their own MM credit or by receiving remittances via MM from relatives and friends. At the health facility level, health workers use a web-based interface to initiate and validate payments by entering a unique transaction authentication number (TAN) and uploading supporting documents such as a photo of the invoice. Semi-automated plausibility checks are performed upon each payment request to protect participants from fraud. Funds remaining on the MMHW are credited to the user’s regular MM account after delivery or at any time prior to completion of the pregnancy if a participant chooses to end her MMHW membership.

#### Restricted cash transfers and electronic vouchers

To incentivize users to save for perinatal medical expenses, each amount credited to the MMHW is topped up with a bonus of 50% of the saved amount. This bonus can only be used to pay for maternal healthcare services; it is not credited to a user’s MM account upon completion of the pregnancy or if a participant chooses to end MMHW membership. In addition, upon sign-up and corresponding to current WHO recommendations, users receive electronic vouchers for free-of-charge (i) ANC drugs such as iron and folic acid supplements; (ii) at least one prenatal ultrasound exam taking place before the 20th week of pregnancy and subsequent follow-up ultrasound exams if medically indicated; and (iii) emergency treatment and referral from a primary-care health facility to a reference hospital in case of a medical emergency during pregnancy or childbirth. Users are informed about their eligibility for conditional cash transfers and electronic vouchers by health workers and automated SMS messages. Users can redeem vouchers for ANC drugs at pharmacies of participating health facilities; ultrasound exams are performed by midwives specialized in prenatal ultrasound at participating health facilities at least once a month, and emergency services can be requested 24/7 by health workers using a Toll-free number. Once a new user has registered for the MMHW and before being eligible to receive restricted cash transfers or electronic vouchers, the account is validated by a facility-based health worker or dedicated MMHW health worker by uploading a photo of the user’s ID card or proof of residence using the MMHW’s web-based interface.

#### Community and home-based procedures

Within both the intervention and control groups of the trial, CHWs will fulfill two functions as part of their routine maternal health activities: (i) to identify pregnant women at the community level during household visits and refer them for ANC or delivery to the nearest primary-care health facility and (ii) to inform pregnant women and their relatives during household visits or community dialogues about general health issues (such as nutrition, immunization, hygiene) and the importance of seeking skilled care during delivery or in case of illness during pregnancy. In the intervention group, CHWs will assume the additional task of informing pregnant women about the MMHW and will encourage them to register for the service. To this end, CHWs will receive information material and training and their feedback will be collected regularly by members of the trial staff. For each user that they sign-up to the MMHW and for each active user of the MMHW, CHWs will receive a small performance-related bonus via MM. However, CHWs in Madagascar’s public health system are involved in a multitude of public health activities and, depending on the type and timing of other interventions, may be unavailable at times for MMHW-related tasks [51]. Thus, in addition to CHWs, dedicated midwives will be employed exclusively for MMHW-related activities to ensure continuous sensitization of pregnant women and relevant stakeholders and to obtain support for the intervention from community leaders. Dedicated health workers will also actively follow up with MMHW users through household visits and phone calls to ensure that they have taken up ANC and to support first and future savings in preparation of delivery through information, education, and counseling. In addition, a toll-free, three-digit hotline providing information and support around MMHW-relevant topics is available to the general public, users, and health facilities.

#### Health facility-based procedures

Health facilities within the intervention group of this study will receive a smart device such as a phone or tablet with mobile internet access and running the MMHW web-based interface and facility-based health workers will be trained in its use. In addition, health workers will also receive training on general aspects of the MMHW enabling them to provide information about the intervention to eligible patients and to collect user feedback. Health facilities within the intervention group will also be invited to take part in a quality assurance program consisting of an annual structured quality assessment followed by a 3-day refresher training for facility-based health workers on routine maternal and newborn care and on complications during pregnancy and childbirth. The quality assessment and refresher training will be performed by representatives of the Ministry of Health. Furthermore, health facilities in the intervention group are entitled to receive small donations of medical equipment including stethoscopes, blood pressure cuffs, weight scales, and sharps disposal containers if deemed necessary by the quality assessment.

#### Control group

The control group obtains the usual standard healthcare and receives: (i) no MMHW; (ii) no conditional cash transfers or electronic vouchers for ANC drugs, prenatal ultrasound examinations, or emergency evacuations; and (iii) no quality of care assessments and trainings for facility-based health workers other than routine activities performed by the Ministry of Health. There are no restrictions on concomitant care during pregnancy in the intervention and control group during the trial, and we recommended to follow the standard of care for Madagascar.

### Data sources

Data definitions and data sources are detailed, separately for primary and secondary outcomes of the trial, in Table 1. Data for the outcome assessment will be drawn from four sources: (i) a quantitative and qualitative population-based survey conducted during household visits, (ii) health facility records, (iii) semi-structured healthcare provider interviews, and (iv) MMHW project dashboard and records. Data collection will be performed using an electronic study database. A data quality manager will check for data entry errors and data inconsistencies on an ongoing basis during the data collection period.

#### Population survey-based impact evaluation

**Fig. 2:**
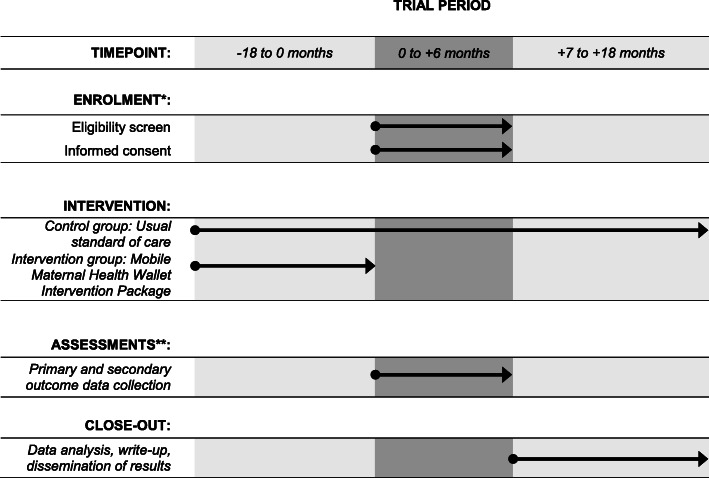
Schedule of enrolment, interventions, and assessments. *Randomization of health-facilities and implementation of the intervention occurred before participant. **Primary and secondary outcome variables and data sources are detailed in Table 1

Data collection will be performed after the conclusion of the intervention period on 01/01/2022 (Fig. 2). The population-based survey will be conducted by a team of trained research assistants with experience in qualitative and quantitative research. Research assistants will be trained and supervised by the study team. All questionnaires contain questions in English with Malagasy translations. The survey will be conducted in Malagasy. The primary sampling unit is a census enumeration area (Malagasy: “fokontany”). We will randomly select one census enumeration area from each of the catchment areas of each public-sector primary-care health facility participating in this trial. Within each census enumeration area, we will then select all households and interview all consenting women meeting the eligibility criteria. This sampling scheme is self-weighting [52]. During the interview, the women’s maternal health booklet will be consulted and photographed to reduce recall bias. During the interviews, we will collect data from two sources: (i) the maternal health booklet, which all women utilizing ANC receive, and (ii) women’s self-report. These two data sources will allow us to measure all of our primary outcomes and will contribute to a mechanistic understanding between exposure and outcomes. In particular, we will extract the data on ANC visits from the maternal health booklet and elicit self-reported data on all outcomes, including ANC visits. In addition, the interviews will elicit data on demographic, behavioral, and socioeconomic factors. Total patient expenditure will be calculated from direct costs (medication, diagnostic, operation during pregnancy, delivery and neonatal period, referral costs) and indirect costs (opportunity costs).

#### Mixed-methods performance evaluation

All 61 health facilities in the control and intervention groups of this trial will be visited by the research team upon the conclusion of the intervention period. Quantitative data on healthcare providers’ service delivery and costs will be drawn from health facility records. Public sector costs will be calculated as a proportion of the resources (i.e., staff, utilities, equipment) directly used by healthcare facilities for maternal and neonatal healthcare services. To achieve a rich and nuanced understanding of the context and assess healthcare providers’ experience, satisfaction, and estimate additional workload introduced by the intervention, semi-structured interviews of consenting healthcare providers will be undertaken at all facilities. Initial interviewees at the health facility will be purposely selected based on their involvement in the intervention (information-rich case sampling), and sampling will be continued via a snowball method in which the initial respondents will be asked to assist in the identification of other respondents who might contribute to the understanding of healthcare provider experience and satisfaction [53]. Interviews will cover themes on service delivery, workload, and costs. Qualitative data collection will continue until saturation and redundancy are reached or financial or logistical constraints necessitate termination of data collection.

#### MMHW project dashboard and implementer records

Data on the intervention costs will be complemented by data from the MMHW dashboard and implementer records. The MMHW dashboard provides data on direct patient-side healthcare uptake and expenditure as well as on conditional cash transfers and electronic vouchers for users and performance-based payments to CHWs. Data on the direct costs of the intervention—including product development, implementation (i.e., hardware), and operation (i.e., hosting and maintenance), will be taken from the implementer’s records.

### Randomization and blinding

**Fig. 3:**
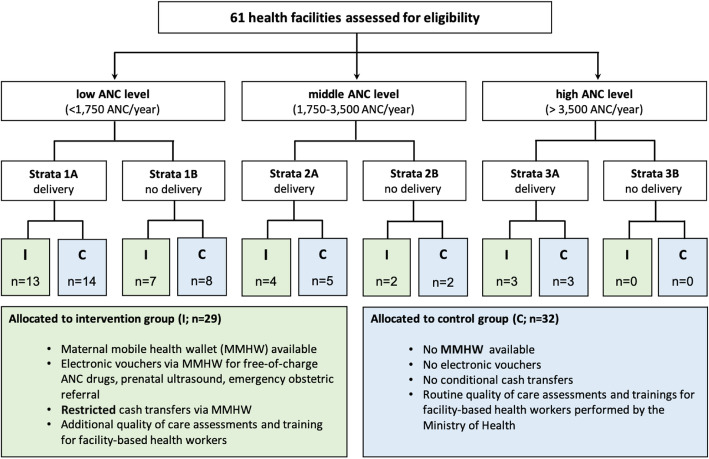
Randomization scheme of the 4MOTHERS trial; I intervention group; C control group

The randomization scheme of this trial is shown in Fig. 3. The unit of randomization for the intervention was one public-sector primary-care health facility. Health facilities in Antananarivo Atsimondrana, Avaradrano, and Renivohitra health districts in the Analamanga region have catchment areas of between 200 and 30,495 inhabitants (mean 3666) and perform 40 to 5438 ANC visits (mean 1556) per year [54, 55]. Randomization of health facilities was performed before the start of the intervention. All 61 public-sector primary-care health facilities in the study region were stratified into 6 subgroups in two stages. In stage one, strata were defined by a health facility’s ANC visit volume (stratum 1: 0–1750, stratum 2: 1750–3500, and stratum 3: >3500 ANC visits per month, respectively). In stage two, strata were defined by the capacity to perform facility-based deliveries (stratum A: no deliveries, stratum B: >1 delivery). Prior empirical evidence indicates that ANC and facility-based delivery quality differ significantly depending on a facility’s patient volume and capacity to perform deliveries [56]. Thus, the sample was stratified by these characteristics to ensure adequate representation of the different kinds of facilities in the final sample. Within each stratum, the facilities were sorted in descending order of the expected number of ANC visits based on the number of reported ANC visits during the year 2017. A pairwise randomization was then applied by a senior biostatistician. The first two health facilities in each stratum were randomly assigned to either the intervention or control group of the study. The algorithm was then repeated until all health facilities were assigned. In addition, four reference hospitals were intentionally assigned to receive the intervention to ensure that women could use the MMHW in case of referral for complications during pregnancy and/or delivery. Due to the nature of the intervention, neither participants nor health facility staff or primary data collectors can be blinded to allocation. Allocation of the intervention will be concealed from outcome assessors including secondary assessors and data analysts.

### Analysis

#### Power calculation

For our baseline power calculation, we assumed a facility-based delivery rate of 67% and an average count of two ANC visits during pregnancy without the intervention [35]. The potential study population was approximately 10,000 pregnant women in the study region during the intervention period. We assumed that 50% of participants living in catchment areas of health facilities that received the intervention would adopt the MMHW. We calculated the minimum detectable difference between the intervention and control groups for the three primary outcomes using methods for cluster randomized trials. We assumed a 20% loss to follow-up and an intra-cluster correlation of 0.05. The intra-cluster coefficient (ICC) was conservative compared to ICCs that were empirically measured in similar settings [57]. Using the standard method for power calculations for binary outcomes, with a 95% two-sided confidence level, we would have 80% power to detect a 4-percentage point increase in the rate of facility-based deliveries, an 80% power to detect an increase of 0.08 average ANC visits per woman, and an increase of total health expenditure of 1700 Malagasy Ariary if we enrolled 2300 participants per group.

#### Analysis of the randomized controlled trial

Our primary analysis will be an intent-to-treat (ITT) causal effect estimation of our registered pre-specified primary and secondary outcomes. The effect sizes determined in the primary analysis provide an unbiased measure of the real-life policy impact of our intervention. As a secondary analysis, we will also conduct a contamination-adjusted intention-to-treat analysis (CA ITT) using an instrumental variable to adjust for bias [58]. For the CA ITT analysis, we will use the random assignment to intervention vs. control facility catchment area of this trial as an instrumental variable (IV) to measure the effect size that would have been attained without “contamination.” Contamination occurs when women who live in an intervention area do not utilize the MMHW and women who live in a control area utilize the MMHW to receive health care. The treatment in the IV analysis is participants’ actual MMHW registration status. The effect size determined in this secondary analysis provides a measure of the full effect that the intervention would have induced in the absence of intervention utilization (or lack thereof) that did not conform with the intervention status intended by the random assignment of geographical communities. We will regress the primary and secondary outcomes (Table 1) on the random assignment of participants to facility catchment intervention vs. control area. For our binary outcomes, we will use modified Poisson regression analysis [59]; for our count outcomes, we will use negative binomial regression; and for our continuous outcomes, we will use linear regression for effect size estimation.

#### Economic evaluation

For the cost-effectiveness analysis, we will empirically quantify the incremental cost-effectiveness ratio (ICER) for the cost per additional facility-based delivery and per additional ANC visit. We will calculate the total healthcare expenditure during pregnancy, delivery, and neonatal period to calculate the incremental costs, which will be divided by the primary outcomes (i) facility-based delivery and (ii) ANC visits. Furthermore, we will calculate ICER with DALYs as denominator by using the standard DALY formula by limiting the disabilities caused by the most common and life-threatening obstetric complications (postpartum hemorrhage, obstructed labor, puerperal sepsis, preeclampsia/eclampsia, and asphyxia) [60–62]. In a second step, we will assess the robustness of the cost-effectiveness analysis by applying a sensitivity analysis. For the quantification of financial distress, we consider the cumulative number of patients reporting at least one of the following signs of financial distress due to maternal and neonatal healthcare costs: (i) assets sold or money borrowed, (ii) relative or friend asked to pay, (iii) catastrophic healthcare expenditure of more than 10% of annual household income, and (iv) essential healthcare forgone because of expected expenditure. All local costs will be calculated in Malagasy Ariary (MGA) before conversion to US dollars (USD) using the average annual exchange rate. Costs and health effects will be discounted and inflation-adjusted using the average consumer price index during the study period. We will use the market price for salary and utility grid to value the additional workload.

#### Data analysis qualitative data

All qualitative interviews will be digitally recorded, verbatim transcribed, and translated by trained scientists. We will adopt in vivo coding, with codes, categories, and themes emerging as we proceed through the data, although the initial coding process will be guided by the specific research questions. This approach will ensure that we pay attention to emerging interpretations of the data, while ensuring fidelity to the initial research question. Strategies to isolate themes will include identifying repetition, typologies, and categories grounded in the data, transitions, similarities and differences, linguistic connectors, missing data, and theory and research question-related material [63]. We will compare information across data sources, checking for consistency in the interpretation. At least two researchers will code and interpret each set of data independently on the basis of a jointly developed and validated codebook. The final interpretation of the findings will be discussed among the entire study team. We will rely on current software applications for support during the coding and analysis process.

#### Data and safety monitoring

To monitor data quality and safety, the aggregate data for each outcome will be analyzed continuously during data collection after the trial period. An independent data and safety monitoring board (DSMB) will assess adherence to the protocol, safety of participants, and progress of the trial as well as data quality and completeness. The board includes two Madagascar- and two Germany-based scientists. In a closed session, the DSMB will be provided with data by the study team’s statistician on each outcome disaggregated by assignment to intervention or control group. All protocol amendments will be communicated directly to the Ethics Committee and trial registries by the project management group, consisting of the principal investigator and the Malagasy study director, as well as the DSMB, and further to the trial participants via the investigators. There are no stopping rules for this trial because it is unlikely that the intervention could lead to adverse effects and the control consists in no additional intervention.

#### Confidentiality

Both primary and secondary data will be stored and handled according to the European Union General Data Protection Regulation guidelines. At the point of data collection, codes based on a series of characteristics will be assigned to participants to maintain participants’ confidentiality. A master sheet linking the code with identifying information, including a name and, when feasible, contact information, will be kept in a secure password-protected online repository that is accessible only to the investigators. Only the principal investigator will have access to and manage this master sheet. The master sheet will facilitate the process of revoking information should a participant decide at a later to have their data removed from the study.

## Discussion

The 4MOTHERS hybrid effectiveness-implementation type-1 trial described in this protocol is the first to determine the causal impact of the MMHW, a mobile money-based savings and payment service, on maternal and neonatal health outcomes and financial access to healthcare. In addition, the trial will gather information on the implementation of the intervention. The quantitative and qualitative research produced from this study will help to understand the economic, social, and behavioral impact of the MMHW in a low-resource public healthcare setting. Furthermore, an economic evaluation will serve to determine the cost-effectiveness of the intervention and to model the costs for its future implementation. Among the strengths of this study is that the intervention was developed based on a human-centered design study harnessing the experiences and recommendations of target communities and key stakeholders by soliciting their feedback on the design of the intervention [41]. Furthermore, the focus on rigorous scientific methodology in a real-world setting including urban, peri-urban, and rural populations and the integration of the trial within the public health system of Madagascar will ensure high applicability and transferability of results to communities in other LMICs. In addition, the rapidly growing penetration of MM services, mobile communication, and other digital technologies and the increasing general tech-savviness in LMICs yield promising pathways for disseminating and scaling the intervention to other under-resourced communities.

The 4MOTHERS trial has several limitations. First, the catchment areas of participating public-sector health facilities selected for the population-based survey are randomly distributed throughout the study region. This creates many boundary areas between intervention and control facilities, which can lead to possible contamination bias should women who live in the catchment area of a control health facility inadvertently be exposed to the intervention, thus potentially minimizing the difference in outcomes between the two groups. Thus, to adjust for contamination bias, we will conduct a CA ITT analysis. Second, the trial takes place in a real-life setting where normal service delivery and targets have to be ensured by participating health facilities alongside the trial intervention, which might impact the adoption of the intervention by health workers. Third, the particular design of the MMHW may impact the uptake and efficiency of the intervention. The trial thus has to be considered as a test of the MMHW “in real life,” and we cannot necessarily conclude from a null finding that mobile money-based savings and payment service for maternal healthcare cannot be effective in another context. Fourth, women who receive the intervention will be entitled to conditional cash transfers and electronic vouchers for free ANC drugs, prenatal ultrasound exams, and emergency evacuation to a reference hospital, which may have an independent effect on maternal and neonatal health outcomes. Therefore, the results of the study can only be considered for the intervention as a whole and should not be reduced to MMHW alone. However, they should be interpreted in the context of effective delivery of conditional cash transfer and electronic vouchers as an inherent strength of the MMHW.

Taken together, we expect that the results of the 4MOTHERS trial will guide future policy decisions and digital health interventions related to improving maternal and neonatal health outcomes and UHC in Madagascar and other countries in SSA. In particular, the results of this trial will inform the Malagasy Ministry of Health and government stakeholders in other countries in SSA whether the integration of the MMHW within national programs is a feasible and beneficial health systems intervention. We will disseminate our results via peer-reviewed journals and presentations at scientific conferences and continuously engage with our policy partners during the operationalization of this trial and as results emerge.

### Trial status

Implementation of the intervention package at all participating public-sector health facilities of the intervention group has been completed in May 2020. The intervention is ongoing. Participants who completed their pregnancy between July 1, 2020, and December 31, 2021, will be enrolled for the randomized outcome assessment starting on January 1, 2022 (Fig. 2). Protocol version: 1.0; issue date: February 15, 2020.

## Supplementary Information

**Additional file 1:.** World Health Organization Trial Registration Data Set.

**Additional file 2:.** Standard Protocol Items: Recommendations for Interventional Trials (SPIRIT) checklist.

**Additional file 3:.** Model consent form.

## Data Availability

The findings of this study will be shared with the Malagasy Ministry of Health. In addition, the full protocol and participant-level anonymized data will be made publicly available using an open data repository. The results of the study will be published in peer-reviewed journals and presented at conferences.

## Abbreviations

4MOTHERS: Madagascar mobile money for maternal healthcare related spending
ANC: Antenatal care
CHW: Community health worker
DALY: Disability-adjusted life year
DSMB: Data and safety monitoring board
GSM: Global system for mobile communication
ICER: Incremental cost-effectiveness ratio
IV: Instrumental variable
LMICs: Low- and middle-income
MGA: Malagasy Ariary
MMHW: Mobile maternal health
MM: Mobile money
OOP: Out-of-pocket
SPIRIT: Standard protocol items: recommendations for interventional trials
SSA: Sub-Saharan Africa
TAN: Transaction authentication number
UHC: Universal health coverage
USD: US Dollars
USSD: Unstructured supplementary service data
WHO: World health organization
