# Supplementary material for: The 4MOTHERS trial of the impact of a mobile money-based intervention on maternal and neonatal health outcomes in Madagascar: study protocol of a cluster-randomized hybrid effectiveness-implementation trial
Source: Trials. 2021 Oct 21;22:725. doi: 10.1186/s13063-021-05694-8 (PMC8529568; doi:10.1186/s13063-021-05694-8)
Supplement: Supplementary file 1 — Additional file 1:. World Health Organization Trial Registration Data Set. [file 13063_2021_5694_MOESM1_ESM.docx]

**Additional file 1.** World Health Organization Trial Registration Data Set.

| **Data category** | [**Information**](http://www.spirit-statement.org/spirit-statement/references#32) |
| --- | --- |
| **Primary registry and trial identifying number** | Deutsches Register Klinischer Studien (German Clinical Trials Register); DRKS00014928 |
| **Date of registration in primary registry** | March 12, 2021 |
| **Secondary identifying numbers** | UTN: U1111-1215-5186 |
| **Source(s) of monetary or material support** | German Federal Ministry of Education and Research (BMBF) |
| **Primary sponsor** | Heidelberg Institute of Global Health University of Heidelberg Im Neuenheimer Feld 130.3, 69120 Heidelberg Germany |
| **Contact for public queries** | Dr Samuel Knauss, MD Charité - Universitätsmedizin Berlin, Charité Platz 1, 10117, Berlin, Germany Email: samuel.knauss@charite.de Phone: +49 (0) 30 450 560 137 |
| **Contact for scientific queries** | Prof. Till Bärnighausen, MD, PhD Heidelberg Institute of Global Health Im Neuenheimer Feld 130.3 69120 Heidelberg Germany Phone: +49 (0) 6221 56-5637  E-Mail: till.baernighausen@uni-heidelberg.de |
| **Scientific title** | Madagascar Mobile Money for MaTernal Healthcare Related Spending (4MOTHERS) |
| **Countries of recruitment** | Madagascar |
| **Health condition(s) or problem(s) studied** | Healthcare financing in reproductive health in Madagascar |
| **Intervention(s)** | Intervention Group: The intervention group has access to the intervention package. The intervention package examined in this study consists of three elements: i) a mobile maternal health wallet for restricted health savings during pregnancy, ii) conditional cash transfers and electronic vouchers for maternal and neonatal healthcare services and emergency evacuation, and iii) quality of care assessments and trainings for facility-based health workers. |
|  | Control Group: The control group obtains the usual standard healthcare and receives: i) no MMHW; ii) no conditional cash transfers or electronic vouchers for ANC drugs, prenatal ultrasound examinations or emergency evacuations; and iii) no quality of care assessments and trainings for facility-based health workers other than routine activities performed by the Ministry of Health. |
| **Key inclusion and exclusion criteria** | Inclusion criteria: Ages eligible for study: ≥18 years Sexes eligible for study: female Additional Inclusion Criteria: Completion of the pregnancy during the intervention period (05/01/2020-10/31/2021). |
|  | Exclusion criteria: younger than 18 years of age, male sex, Completion of the pregnancy outside of the study period. |
| **Characteristics** | Study Type: Interventional Allocation: Randomized controlled trial Design: Hybrid effectiveness-implementation type-1 trial Intervention model: Parallel assignment Blinding: Blinded outcome assessment Primary purpose: Health care system |
| **Date of first enrolment** | 11/01/2021 |
| **Target sample size** | 4600 |
| **Recruitment status** | not yet recruiting |
| **Primary outcome(s)** | 1) Facility-based delivery (woman delivering at a health facility)  2) Antenatal care visits (ANC visits at a health facility per woman)  3) Total healthcare expenditures (total health expenditures during pregnancy, delivery and neonatal period per woman) |
| **Key secondary outcomes** | 1) ANC diagnoses (diagnoses detected during ANC per woman) 2) Complications (pregnancy or childbirth-related complications) 3) Postpartum depression (Women interviewed after delivery fulfilling the screening criteria for depression) 4) Maternal mortality 5) Newborn mortality  6) Third parties’ financial contributions (Funds received from relatives and friends for maternal and neonatal healthcare) 7) Relative healthcare expenditures (Ratio of total healthcare expenditures of household income) 8) Public sector costs (Healthcare expenditures per woman during pregnancy, delivery and neonatal period) 9) Cost per additional facility-based delivery (Cost-effectiveness ratio: effect on the first primary endpoint divided by effect on the third primary endpoint) 10) Cost per additional ANC visit (Cost-effectiveness ratio: effect on the second primary endpoint divided by effect on the third primary endpoint 11) Financial distress (Woman reporting at least one sign of financial distress) 12) Time to seek medical attention (Time from first symptoms until professional medical care was accessed) 13) MMHW usage (Woman who used the MMHW to pay for ANC or delivery-related expenses either in part or entirely) 14) Patient satisfaction (Patient satisfaction with health facility and MMHW) 15) Health system satisfaction (Satisfaction with the health system) |
|  |  |
