# Supplementary material for: The 4MOTHERS trial of the impact of a mobile money-based intervention on maternal and neonatal health outcomes in Madagascar: study protocol of a cluster-randomized hybrid effectiveness-implementation trial
Source: Trials. 2021 Oct 21;22:725. doi: 10.1186/s13063-021-05694-8 (PMC8529568; doi:10.1186/s13063-021-05694-8)
Supplement: Supplementary file 3 — Additional file 3:. Model consent form. [file 13063_2021_5694_MOESM3_ESM.pdf]

Consent Form „Work Package 1”
„Household Visit Survey – Impact“

Study Title:
Madagascar Mobile MOney for MaTernal HEalthcare Related Spending (4MOTHERS) trial in Madagascar

Study Team:
Institute of Public Health, University of Heidelberg, Germany, Doctors for Madagascar (NGO), Antananarivo, Madagascar

Relevant Contact:
Doctors for Madagascar, Pres II Y 53bis, Rue Dr Rabenoro, Andrainarivo, Antananarivo

I declare that I have been verbally informed of all the details of the study. In addition, I received a written copy of the information sheet.
I declare that I was given the opportunity to ask questions and that all my questions received satisfactory answers.
I declare that it was clarified to me and that **I agree with the fact that the information collected from me for this study will be stored, used, and potentially forwarded to others pseudonymized. Third parties will at no point in time have access to my personal information. My name will not be disclosed during the process of publishing the results of the study.**
I declare that my participation in this study is voluntary. I reserve the right to withdraw from the study and/or to withdraw the material collected from me at any point in time.

Respondent's name (Family Name, First Name):

……………………………………………………………………………………

Respondent's signature and date:

……………………………………………………………………………………
